# Supplementary material for: Revealing the Arabidopsis AtGRP7 mRNA binding proteome by specific enhanced RNA interactome capture
Source: BMC Plant Biol. 2024 Jun 14;24:552. doi: 10.1186/s12870-024-05249-4 (PMC11177498; doi:10.1186/s12870-024-05249-4)
Supplement: Supplementary file 2 — Supplementary Material 2 [file 12870_2024_5249_MOESM2_ESM.pdf]

ADDITIONAL FILE 2

Figure S1: Uncropped blots to Fig. 1D

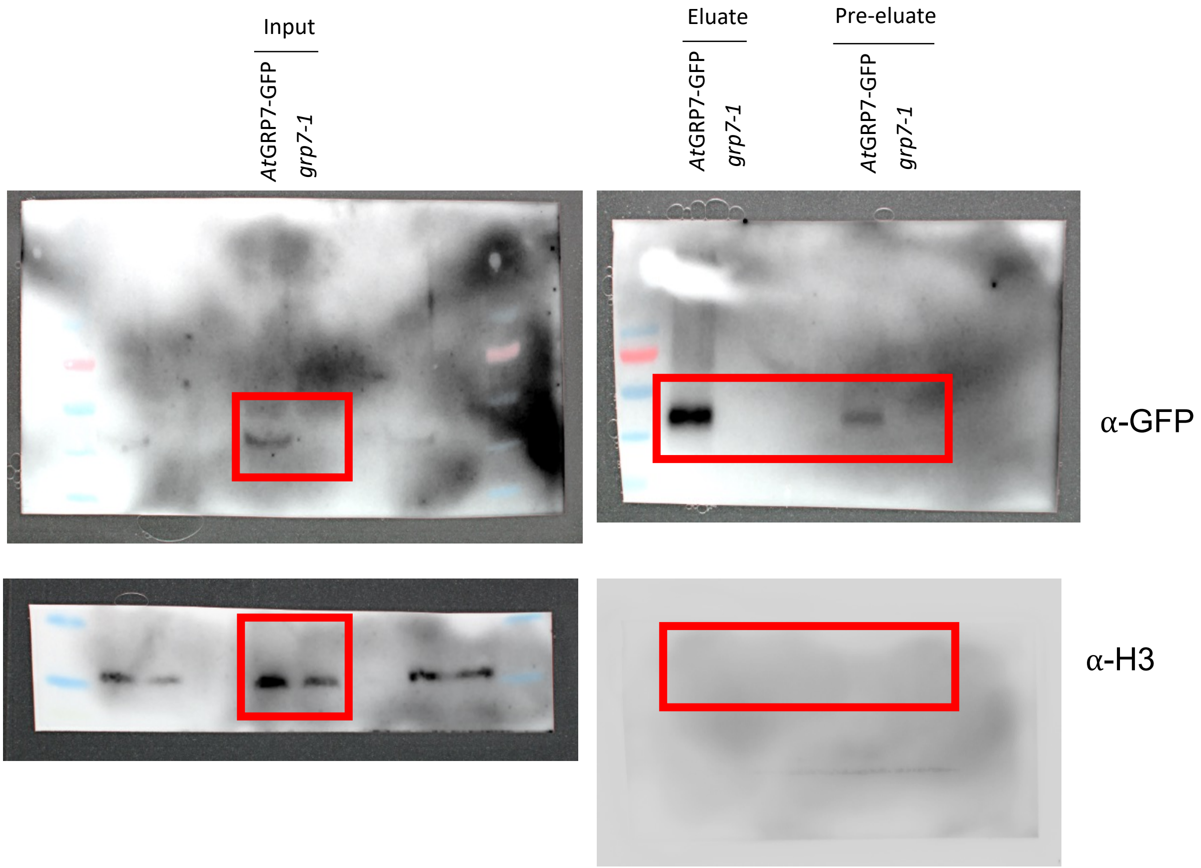

Immunoblot analysis of *AtGRP7*::*At*GRP7-GFP *grp7-1* and *grp7-1* control plants subjected to RNP capture with 5'UTR_1 LNA oligonucleotides.

Figure S2: Uncropped gels to Additional file 3C and 3D

3C

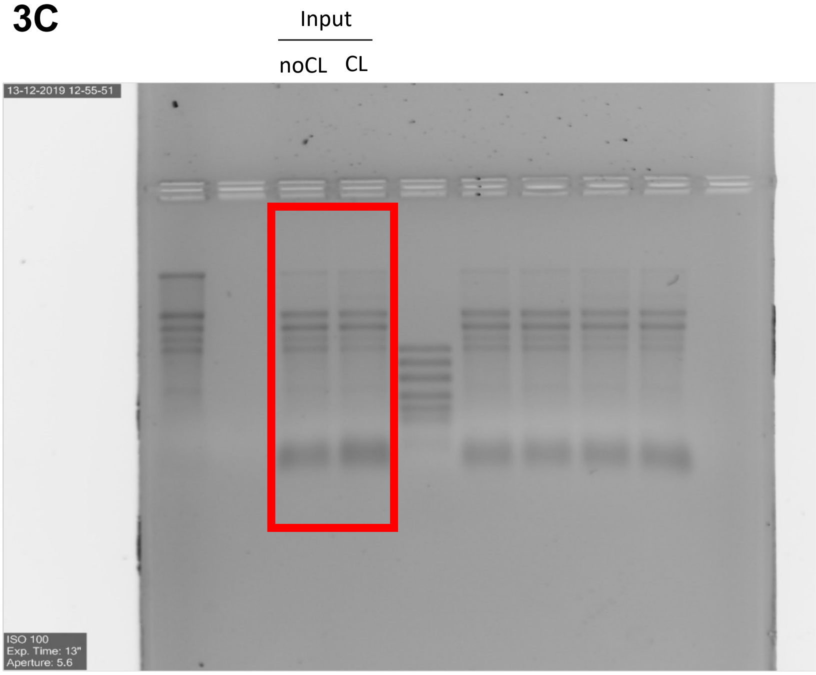

3D

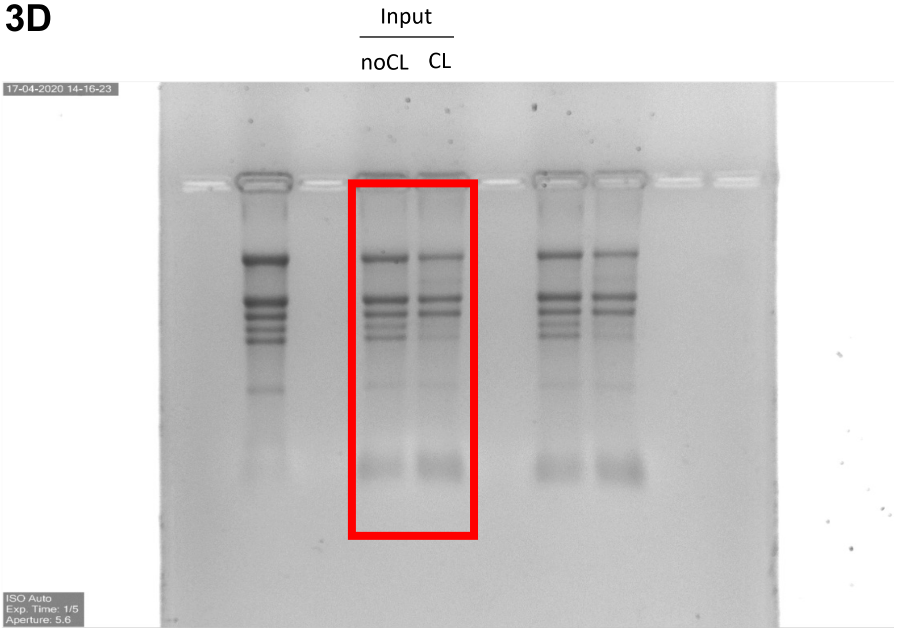

Agarose-formaldehyde gel electrophoresis of total RNA in the input of crosslinked and non-crosslinked samples after tandem capture with the LNA 5'UTR_1 probe followed by oligo(dT) capture using the protocol of Rogell *et al.*, 2017 **(3C)** or Chu *et al.*, 2015 **(3D)**.

Figure S3: Uncropped gel to Additional file 4B

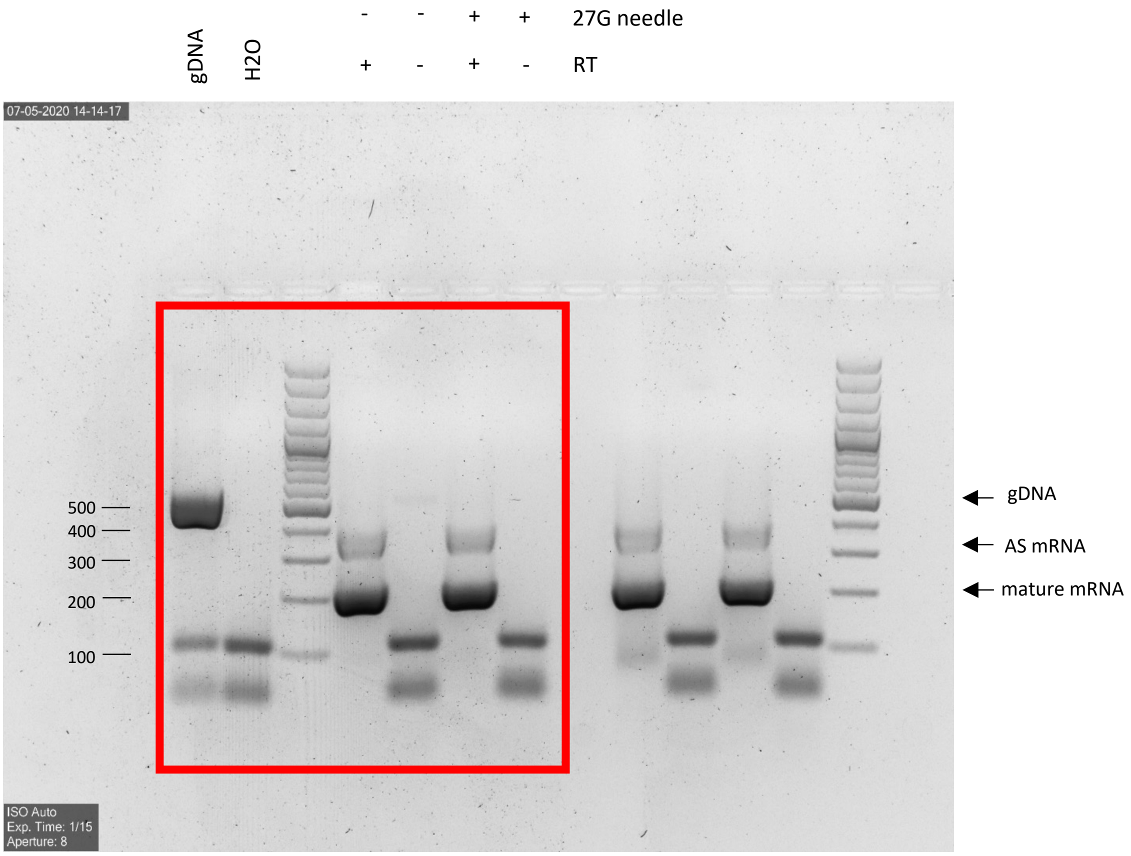

Comparison of the level of genomic *At*GRP7 DNA in the cell lysate without passage (-27G needle) or after passage through a 27G needle (+27G needle). RT-PCR was performed with (+RT) or without (-RT) prior reverse transcription. For comparison, amplification from genomic DNA (gDNA) is shown. Arrows indicate the amplicons derived from gDNA, alternatively spliced pre-mRNA and the fully spliced mRNA.

Figure S4: Uncropped gels to Fig. 3A and Fig. 3B

3A

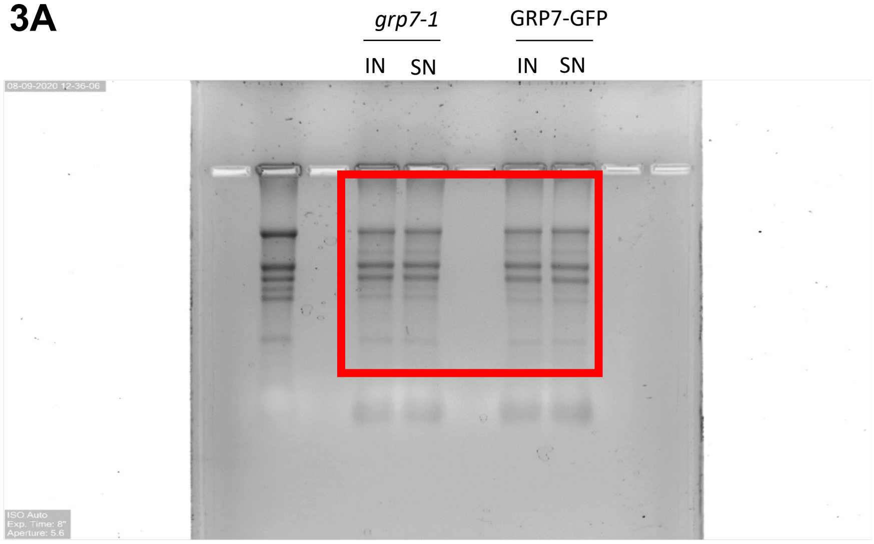

Agarose-formaldehyde gel electrophoresis of total RNA in the lysate (input) and the supernatant after probe hybridization (SN) in *AtGRP7*-GFP *grp7-1* plants and *grp7-1* control plants.

3B

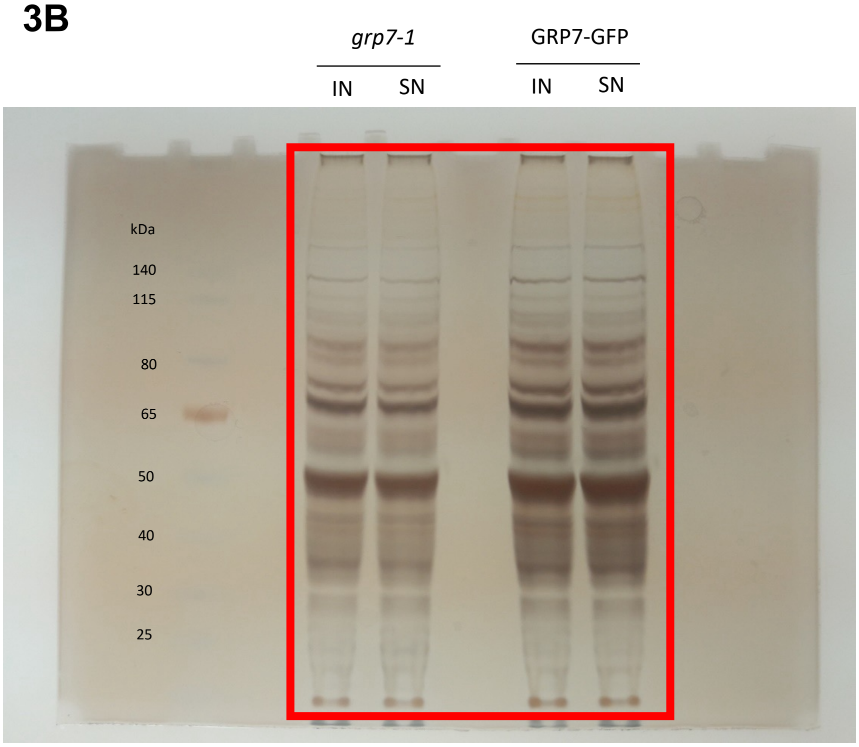

Silver staining of total protein in the lysate (input) and the supernatant after probe hybridization (SN) in the *AtGRP7*-GFP *grp7-1* plants and *grp7-1* control plants.

Figure S5: Uncropped gels to Additional file 6A and 6B

6A

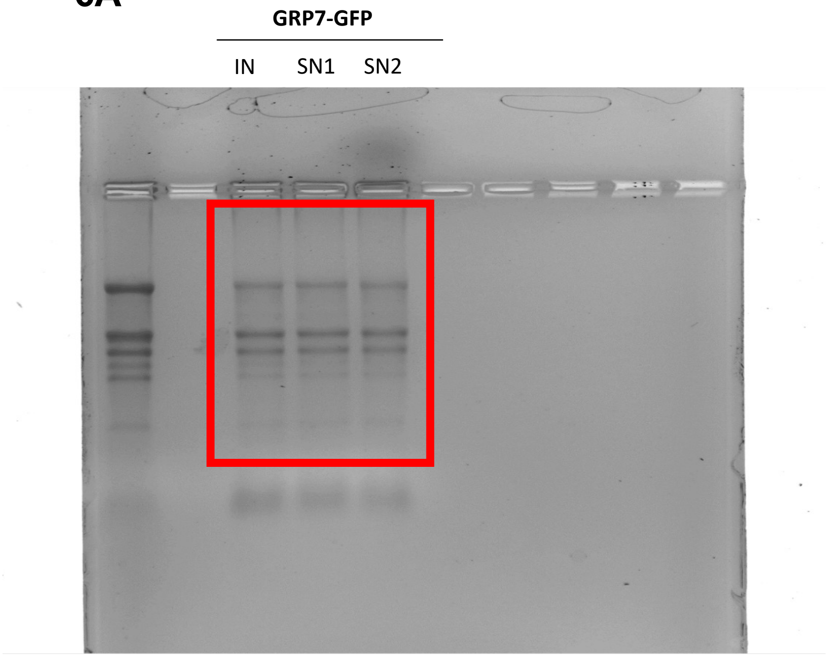

Agarose-formaldehyde gel electrophoresis of total RNA in the lysate (input) and the supernatant after the first round (SN1) and second round (SN2) of probe hybridization in the *AtGRP7*-GFP *grp7-1* plants.

6B

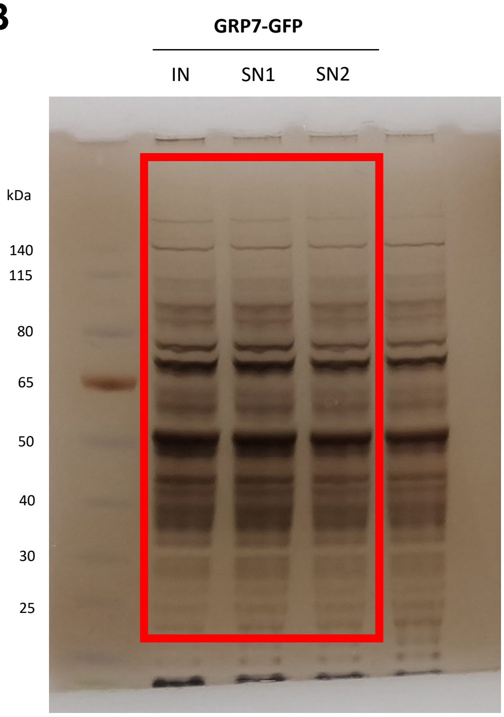

Silver staining of total protein in the lysate (input) and the supernatant after the first round (SN1) and second round (SN2) of probe hybridization in the *AtGRP7*-GFP *grp7-1* plants.

Figure S6: Uncropped gels to Fig. 6A, 6B, 6C and 6D

6A

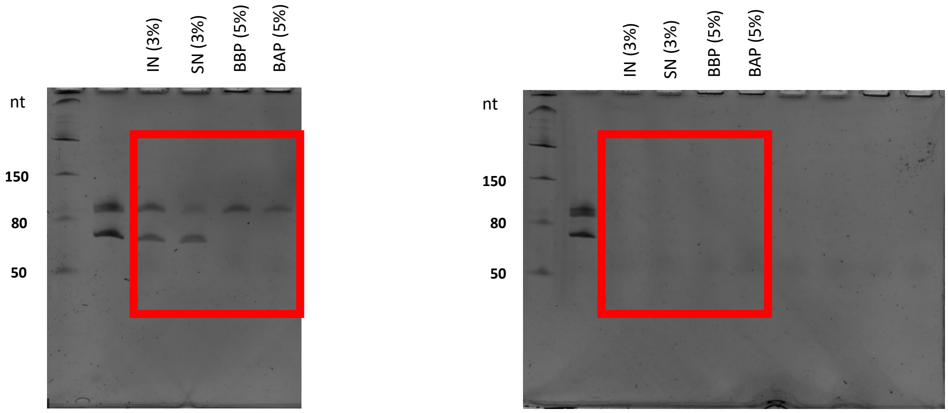

5’UTR bait

Empty beads

6B

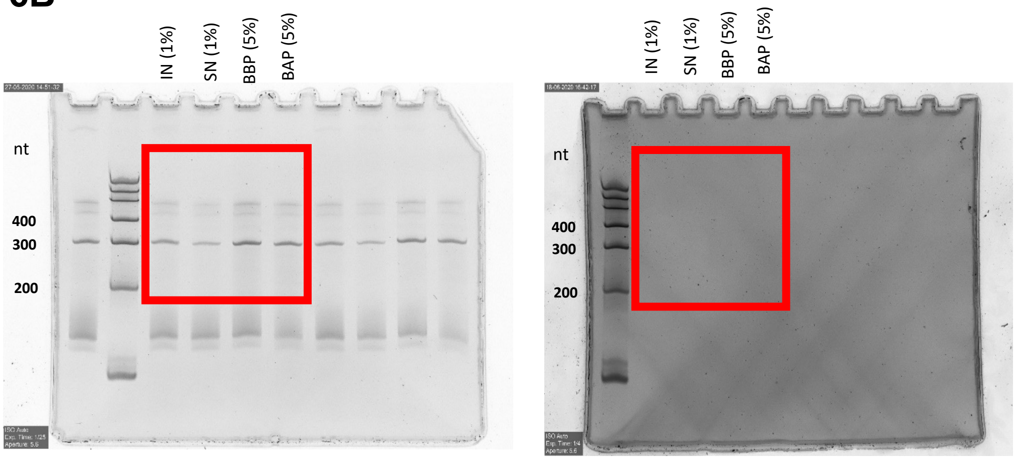

3’UTR bait

Empty beads

Coupling efficiency of biotinylated 5’UTR bait (**6A**) and 3’UTR bait (**6B**) to magnetic streptavidin beads. Aliquots of the RNA isolated from the input (IN), supernatant after coupling (SN) and eluates from the beads before (BBP) and after pulldown (BAP) were analyzed on 12.5% urea PAGE gels. Empty beads were used as controls.

6C

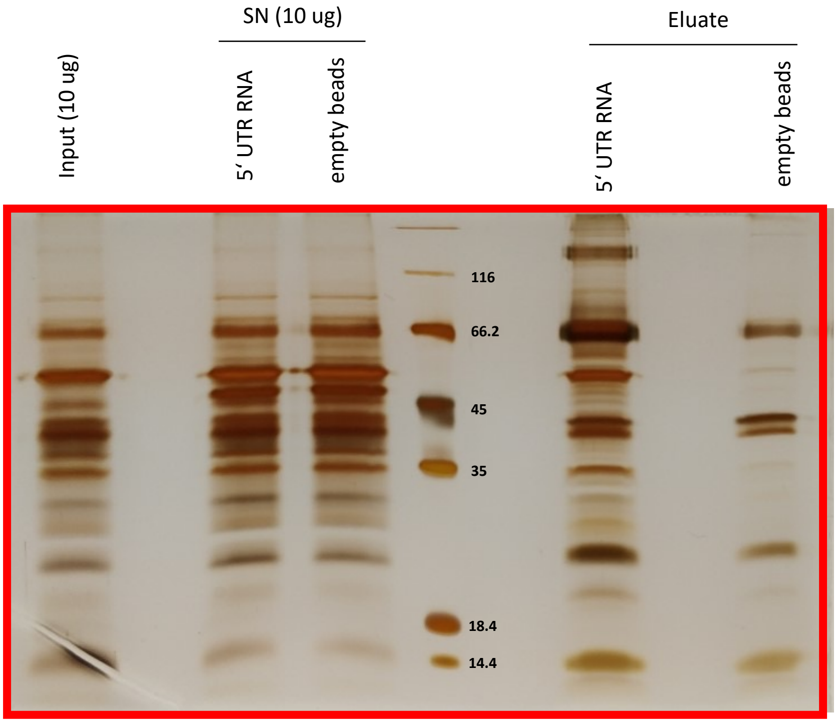

6D

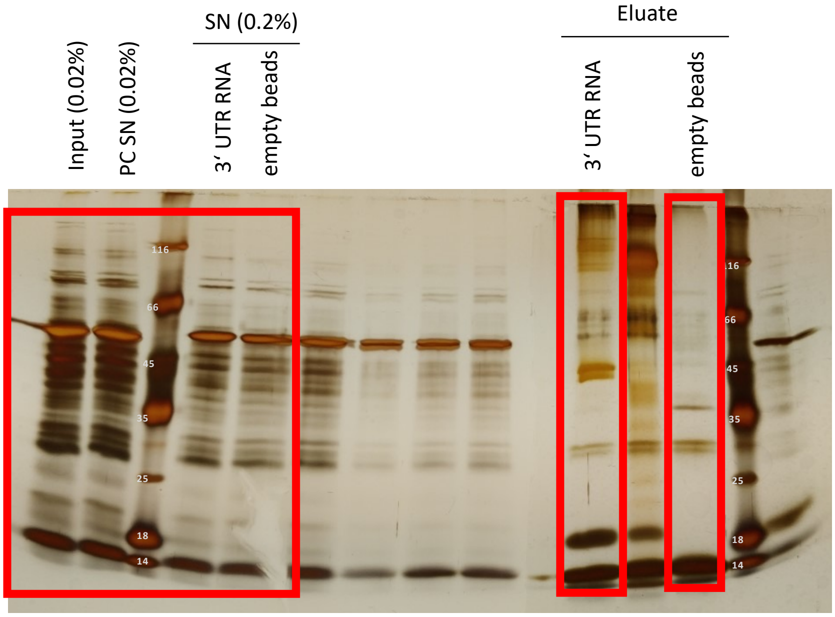

Silver staining of proteins recovered after *in vitro* captures with 5’UTR bait (**6C**) and 3’UTR bait (**6D**). Aliquots of the input, supernatant and eluates of the respective coupled and empty beads were separated on a 12% SDS-Page and subjected to silver staining.

Figure S7: Uncropped gel to Additional file12

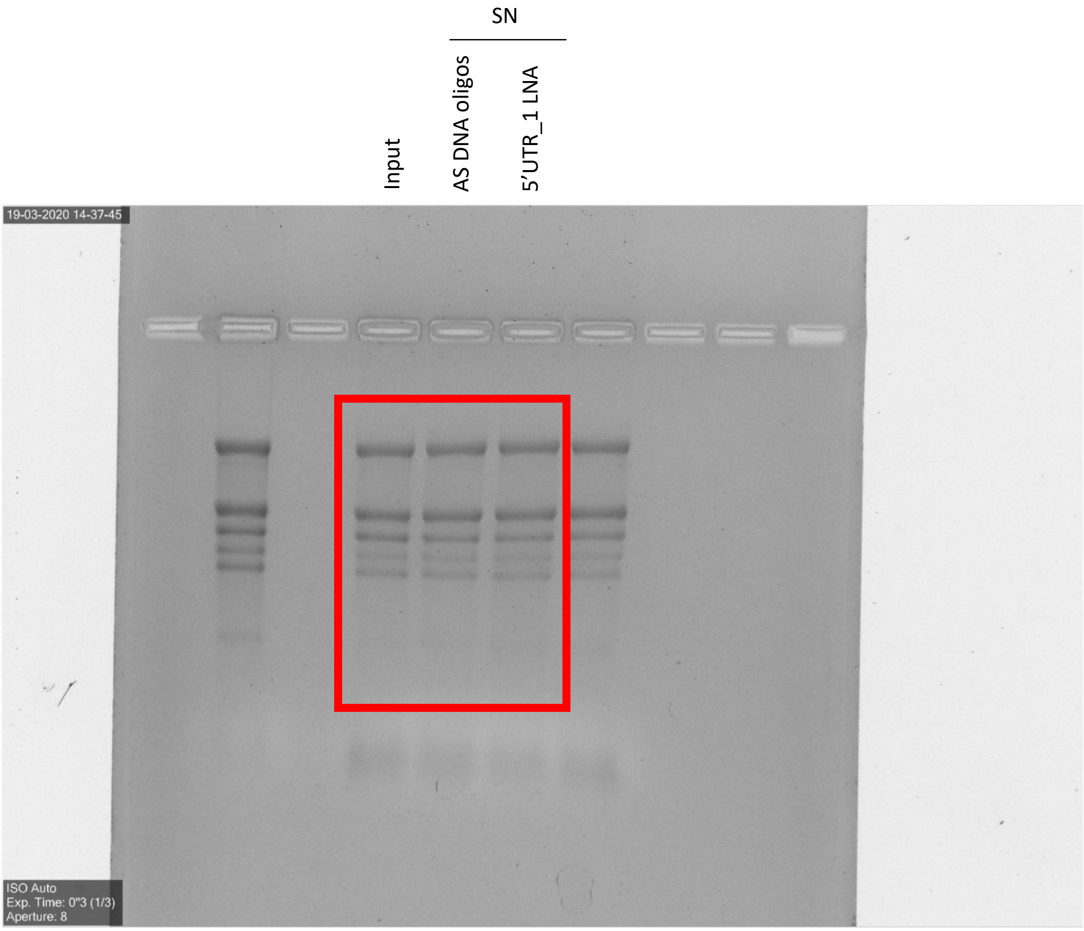

Agarose-formaldehyde gel electrophoresis of total RNA in the lysate (input) and the supernatant (SN) after hybridization with AS DNA oligonucleotides or the 5'UTR_1 LNA oligo in *AtGRP7*-GFP *grp7-1* plants.
