# Supplementary material for: Revealing the Arabidopsis AtGRP7 mRNA binding proteome by specific enhanced RNA interactome capture
Source: BMC Plant Biol. 2024 Jun 14;24:552. doi: 10.1186/s12870-024-05249-4 (PMC11177498; doi:10.1186/s12870-024-05249-4)
Supplement: Supplementary file 3 — Supplementary Material 3 [file 12870_2024_5249_MOESM3_ESM.pdf]

Additional file 3

(A) Protocol based on Rogell *et al.* (2017)

Step	Buffer composition	Procedure
Lysis	20 mM Tris-HCl pH7.5, 500 mM LiCl, 0.5% LiDS, 4mM $MgCl_2$, 0.02% NP40<br>Fresh: 5mM DTT, 2.5% PVP40 1% β-ME, 1x Complete Protease Inhibitor, 10 mM RVC, 15% formamide (added before hybridization)	40 nmol 5'UTR LNA probe was added to 50 ml lysate prepared from 8 g ground plant tissue; probe hybridization at 4°C for 2h on rotator
Washes	**Buffer I:** 20 mM Tris-HCl pH7.5, 500 mM LiCl, 0.1% LiDS, 4mM $MgCl_2$, 0.02% NP40, 5 mM DTT<br>**Buffer II**: 20 mM Tris-HCl pH7.5, 500 mM LiCl, 4mM $MgCl_2$, 0.02% NP40, 5 mM DTT<br>**Buffer III**: 20 mM Tris-HCl pH7.5, 200 mM LiCl, 4mM $MgCl_2$, 5 mM DTT	2 washes with each buffer for 5 min at 4°C
Pre-elution	H_2O	40°C, 5 min, 800 rpm
Elution	10 mM Tris-HCl pH7.5, 1 mM EDTA	90°C, 3 min, 800 rpm

(B) Protocol based on Chu *et al.* (2015)

Step	Buffer composition	Procedure
Lysis	50 mM Tris-HCl pH 7.5, 500 mM NaCl, 0.5% SDS, 1 mM EDTA<br>Fresh: 5 mM DTT, 2.5% PVP40, 1% beta-ME, 1x Complete Protease Inhibitor, 15% formamide (added before hybridization)	40 nmol 5'UTR LNA probe was added to 50 ml lysate prepared from 8 g ground plant tissue; probe hybridization at 4°C for 2h on rotator
Washes	**Buffer I**: 2x SSC pH 7.0, 0.5% SDS, 5 mM DTT<br>**Buffer II**: 1x with 1x SSC pH 7.0	4 washes with buffer I<br>1 wash with buffer 2 for 5 min at RT
Pre-elution	1x SSC pH 7.0	40°C, 5 min, 800 rpm
Elution	10 mM Tris-HCl pH7.5, 1 mM EDTA	90°C, 3 min, 800 rpm

(C)

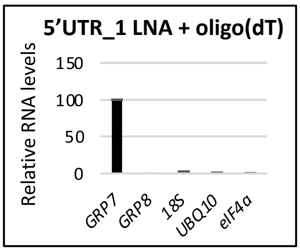

Transcript	C_T value
GRP7	16.48
GRP8	29.24
18S	21.76
UBQ10	22.98
eIF4a	24.04

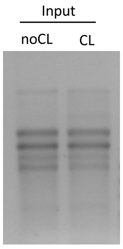

(D)

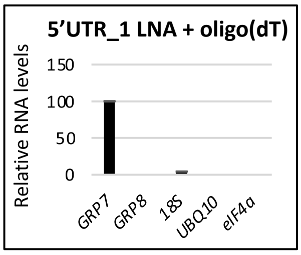

Transcript	C_T value
GRP7	15.61
GRP8	30.69
18S	20.14
UBQ10	22.48
eIF4a	22.45

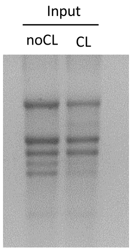

Additional file 3: Experimental procedure and composition of the buffers used for optimization of the specific RNP capture.

(A, B) Buffer composition and protocol based on Rogell *et al.* (2017) for specific capture of rRNA binding proteins **(A)** and Chu *et al.* (2015) for ChIRP-MS **(B)**. **(C, D)** Analysis of RNA levels in eluates determined by RT-qPCR (left), corresponding C_T values (middle), and agarose-formaldehyde gel electrophoresis of total RNA in the input of crosslinked and non-crosslinked samples (right) after tandem capture with the LNA 5'UTR_1 probe followed by oligo(dT) capture using the protocol of Rogell *et al.*, 2017 **(C)** or Chu *et al.*, 2015 **(D)**.
