# Supplementary material for: Revealing the Arabidopsis AtGRP7 mRNA binding proteome by specific enhanced RNA interactome capture
Source: BMC Plant Biol. 2024 Jun 14;24:552. doi: 10.1186/s12870-024-05249-4 (PMC11177498; doi:10.1186/s12870-024-05249-4)
Supplement: Supplementary file 4 — Supplementary Material 4 [file 12870_2024_5249_MOESM4_ESM.pdf]

Additional file 4

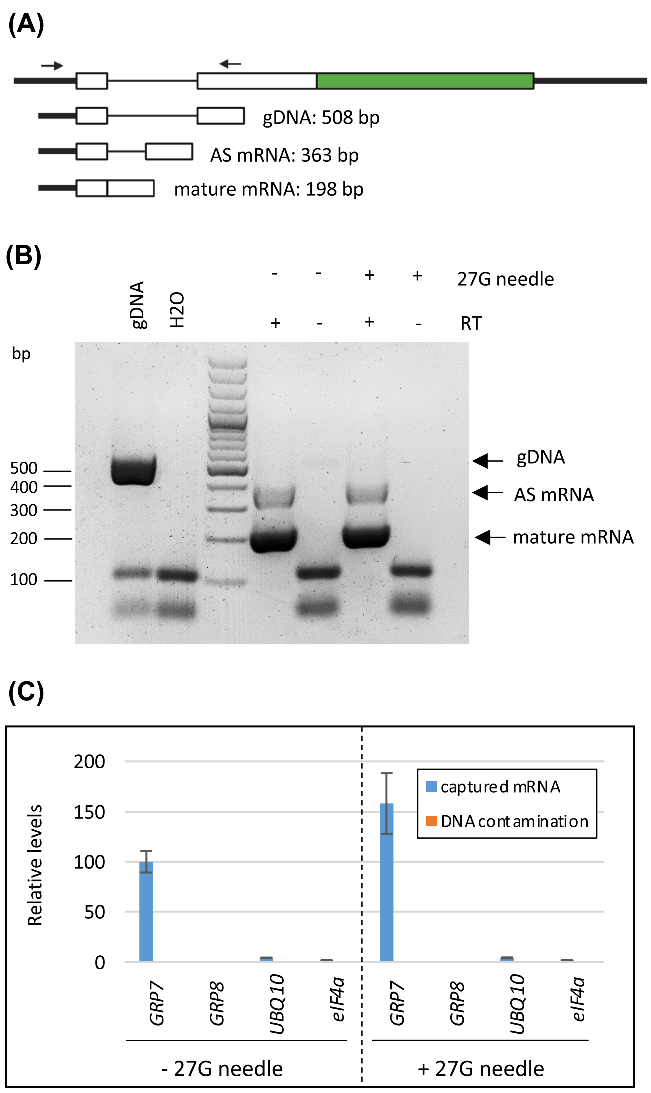

Additional file 4: Removal of residual genomic DNA by shearing.

(A) Schematic diagram of primers spanning the *AtGRP7* intron and amplicons from genomic DNA (gDNA), alternatively spliced (AS) pre-mRNA and mature mRNA. **(B)** Comparison of the level of genomic *At*GRP7 DNA in the cell lysate without passage (-27G needle) or after passage through a 27G needle (+27G needle). RT-PCR was performed with (+RT) or without (-RT) prior reverse transcription. For comparison, amplification from genomic DNA (gDNA) is shown. Arrows indicate the amplicons derived from gDNA, alternatively spliced pre-mRNA and the fully spliced mRNA [42]. **(C)** Relative levels of *AtGRP7*, *AtGRP8*, *UBIQUITIN 10*, and *eIF4a* mRNA (blue) or gDNA contamination (orange) in eluates without passage (-27G needle) or after passage of the lysate through a 27G needle (+27G needle). The *AtGRP7* level is set to 100 %.
