# Supplementary material for: Revealing the Arabidopsis AtGRP7 mRNA binding proteome by specific enhanced RNA interactome capture
Source: BMC Plant Biol. 2024 Jun 14;24:552. doi: 10.1186/s12870-024-05249-4 (PMC11177498; doi:10.1186/s12870-024-05249-4)
Supplement: Supplementary file 6 — Supplementary Material 6 [file 12870_2024_5249_MOESM6_ESM.pdf]

Additional file 6

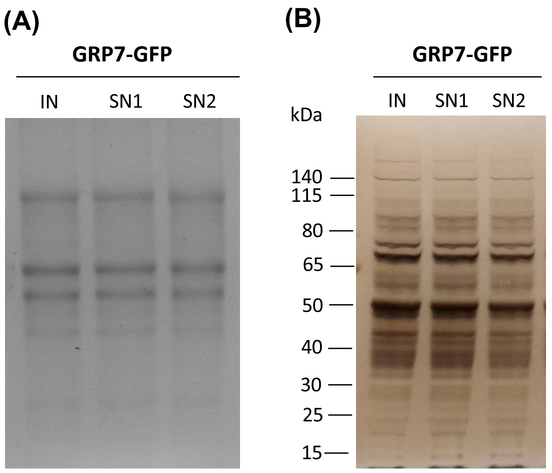

(C)

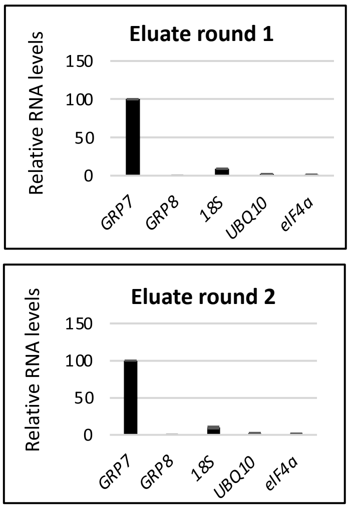

Transcript	C_T value
GRP7	16.56
GRP8	28.35
18S	20.03
UBQ10	22.12
eIF4a	22.82

Transcript	C_T value
GRP7	17.12
GRP8	28.56
18S	20.49
UBQ10	22.44
eIF4a	23.11

Additional file 6: Protein and RNA analysis upon two rounds of capture with 5'UTR_1 LNA oligo.

(A) Agarose-formaldehyde gel electrophoresis of total RNA in the lysate (input) and the supernatant after the first round (SN1) and second round (SN2) of probe hybridization in the *AtGRP7*-GFP *grp7-1* plants. **(B)** Silver staining of total protein in the lysate (input) and the supernatant after the first round (SN1) and second round (SN2) of probe hybridization in the *AtGRP7*-GFP *grp7-1* plants. The positions of the molecular weight markers are indicated. **(C)** Relative *AtGRP7*, *AtGRP8*, 18S rRNA, *UBIQUITIN10*, and *eIF4a* RNA levels in the eluates of the first round (top) and the second round (bottom). Right, corresponding CT values.
