# Supplementary material for: Revealing the Arabidopsis AtGRP7 mRNA binding proteome by specific enhanced RNA interactome capture
Source: BMC Plant Biol. 2024 Jun 14;24:552. doi: 10.1186/s12870-024-05249-4 (PMC11177498; doi:10.1186/s12870-024-05249-4)
Supplement: Supplementary file 7 — Supplementary Material 7 [file 12870_2024_5249_MOESM7_ESM.pdf]

# Additional file 7

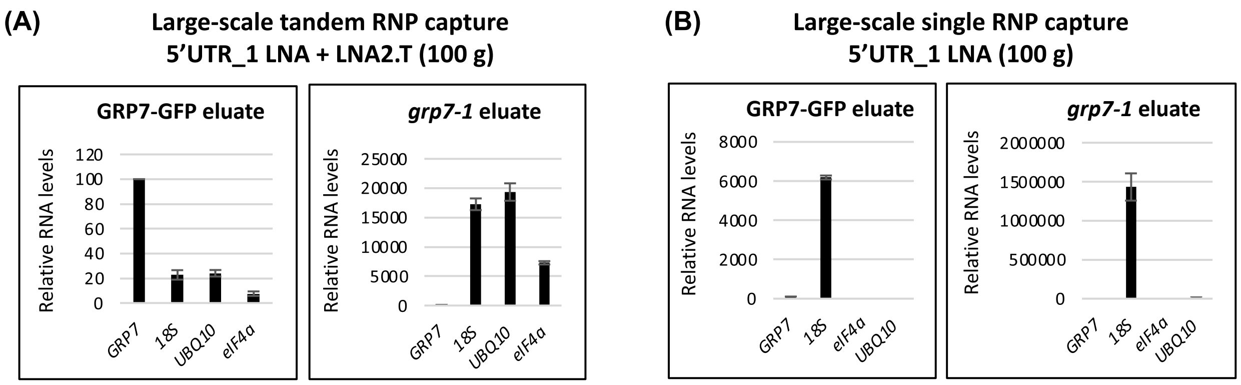


**Additional file 7: RNA levels in the eluates after large-scale captures.**

Relative *AtGRP7*, 18S rRNA, *UBIQUITIN 10*, and *eIF4α* RNA levels in the eluates of *AtGRP7*-GFP *grp7-1* plants (left) and *grp7-1* control plants (right) upon large scale tandem capture with two consecutive rounds of hybridization with the 5’UTR_1 LNA oligonucleotide followed by LNA2.T capture **(A)**, or large-scale single capture with two consecutive rounds of hybridization with the 5’UTR_1 LNA oligo only **(B)**.
