# Supplementary material for: Revealing the Arabidopsis AtGRP7 mRNA binding proteome by specific enhanced RNA interactome capture
Source: BMC Plant Biol. 2024 Jun 14;24:552. doi: 10.1186/s12870-024-05249-4 (PMC11177498; doi:10.1186/s12870-024-05249-4)
Supplement: Supplementary file 8 — Supplementary Material 8 [file 12870_2024_5249_MOESM8_ESM.pdf]

Additional file 8

AGI	TAIR alias
**RRM domain proteins**	
AT1G01080	CP28B
AT1G03457	BRN2
AT2G21660	GRP7
AT2G32080	PUR-ALPHA-1
AT5G54900	RBP45A
AT4G27000	RBP45C
AT5G02530	ALY2
AT3G52380	CP33A
AT2G35410	CP33B
AT4G09040	CP33C
**Ribosomal proteins**	
AT1G35680	RPL21C
AT2G36160	US11Z
AT3G04920	ES24Z
AT5G15200	US4Z
AT5G16130	ES7X
**Translation factors**	
AT3G11400	EIF3G1
AT3G13920	EIF4A1 (RH4)
**ALBA proteins**	
AT1G20220	ALBA2
AT1G76010	ALBA4
**Nuclear Transport proteins**	
AT3G25150	ATG3BP-3
AT5G60980	ATG3BP-1
**Others**	
AT1G48920	NUC1
AT5G26742	RH3
AT5G63260	ATC3H67
AT4G29060	EMB2726
AT4G16390	SVR7
AT5G04430	ATKH22
AT4G28440	
AT3G23700	SRRP1
ATCG00490	RBCL

Additional file 8: **List of 30 common proteins** from the large-scale single capture with two consecutive rounds of hybridization with the 5’UTR_1 LNA oligonucleotide and the large-scale tandem capture with hybridization with 5’UTR_1 LNA oligonucleotide followed by LNA2.T capture.
