# Supplementary material for: Revealing the Arabidopsis AtGRP7 mRNA binding proteome by specific enhanced RNA interactome capture
Source: BMC Plant Biol. 2024 Jun 14;24:552. doi: 10.1186/s12870-024-05249-4 (PMC11177498; doi:10.1186/s12870-024-05249-4)
Supplement: Supplementary file 9 — Supplementary Material 9 [file 12870_2024_5249_MOESM9_ESM.pdf]

Additional file 9

(A)

In vitro transcript for *GRP7* 5'UTR capture

T7 promoter | 5'UTR | Exon 1

+1

5' - taatacgactcactatagg**GTCTACATCGTTCTACACATCTCACTGCTCACTACTCTCACTGTAATCCCTTAGATCTTCTTTTCAAATTTCA**ATGGCGTCCGGTGAT – 3'

TSS

(B)

In vitro transcript for *GRP7* 3'UTR capture

T7 promoter | 3'UTR

5' - taatacgactcactataggGGGTACC**TTCCTTTAATTAGGTTTGGGATTACCAATGAATGTTCTCTCTCTCGCTTGTTATGCTTCTACTTGGTTTTGTGTGTTCTCTAT**
TTTGTTCTGGTTCTGCTTTAGATTTGATGTAACAGTTCGTGATTAGGTATTTTGGTATCTGGAAACGTAATGTTAAGTCACTTGTCATTCTCTAAATAACAAATTT
CTTCGGAGATATTATCTCTGTTGATTGATTCTATCATCTAAATTTCGGAAATGCTAATCGGATACCGGAATGGTGCCTTTGTTACGATTGAGTTACGGATCC – 3'

Additional file 9: **Sequences of baits** derived from the *AtGRP7* 5'UTR **(A)** and 3'UTR **(B)** used for *in vitro* pulldowns.
