# Supplementary material for: Revealing the Arabidopsis AtGRP7 mRNA binding proteome by specific enhanced RNA interactome capture
Source: BMC Plant Biol. 2024 Jun 14;24:552. doi: 10.1186/s12870-024-05249-4 (PMC11177498; doi:10.1186/s12870-024-05249-4)
Supplement: Supplementary file 10 — Supplementary Material 10 [file 12870_2024_5249_MOESM10_ESM.pdf]

Additional file 10

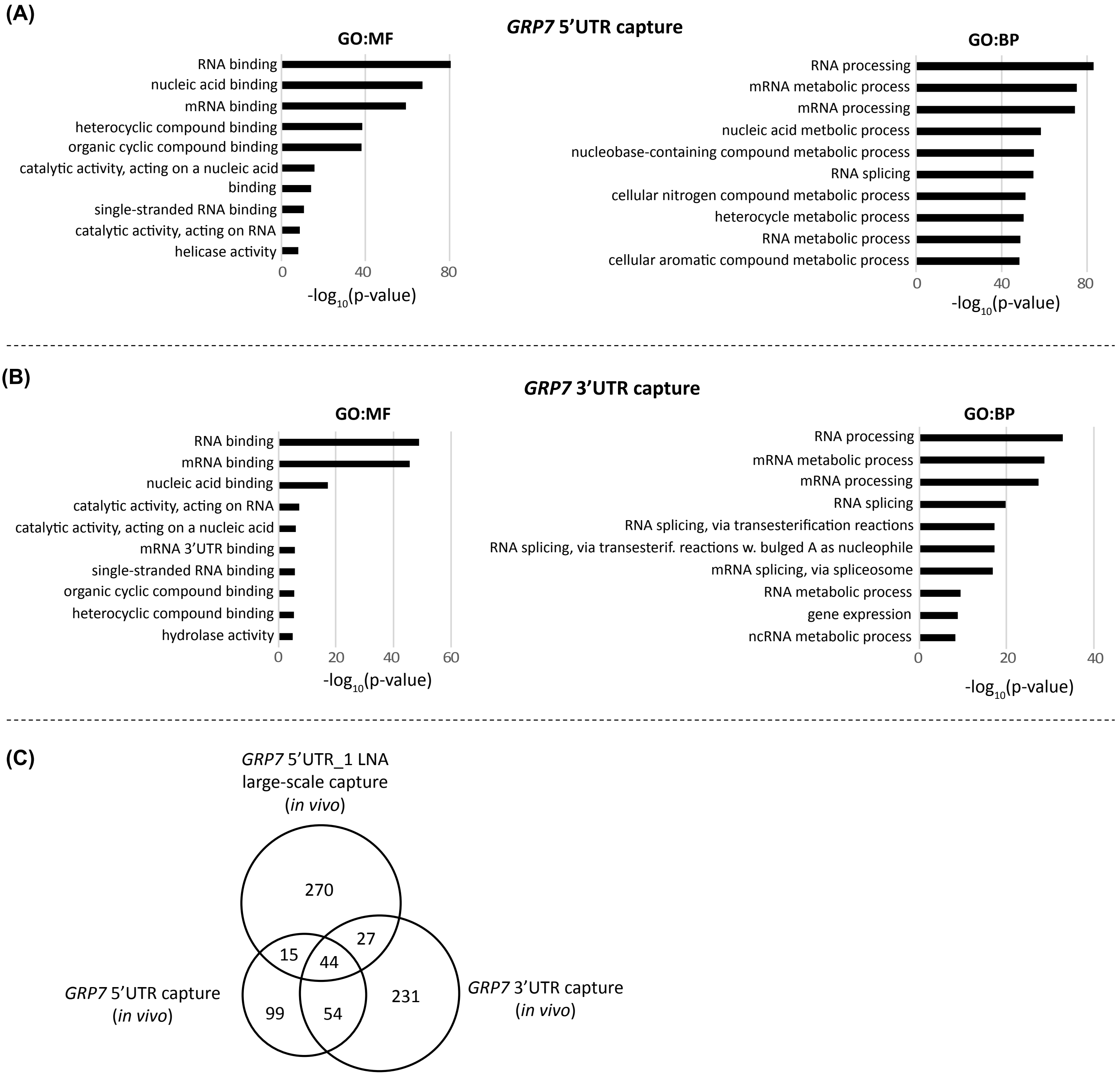

Additional file 10:**GO term analysis of proteins identified by *in vitro* captures**

Enriched GO terms for molecular function and biological process for proteins significantly enriched after *in vitro* capture with the 5'UTR bait **(A)** and 3'UTR bait **(B)**. **(C)** Venn diagram of proteins identified by large scale *in vivo* capture with the 5'UTR_1 LNA oligo and significantly enriched proteins from the *in vitro* pulldowns.
