# Supplementary material for: Revealing the Arabidopsis AtGRP7 mRNA binding proteome by specific enhanced RNA interactome capture
Source: BMC Plant Biol. 2024 Jun 14;24:552. doi: 10.1186/s12870-024-05249-4 (PMC11177498; doi:10.1186/s12870-024-05249-4)
Supplement: Supplementary file 11 — Supplementary Material 11 [file 12870_2024_5249_MOESM11_ESM.pdf]

Additional file 11

Translation factors

AGI	TAIR alias	*in vivo (large-scale)* *GRP7 5'UTR_1 LNA*	*in vivo (large-scale)* *GRP7 5'UTR_1 LNA + LNA2.T*	*in vitro* *5'UTR*	*in vitro* *3'UTR*
AT3G11400	EIF3G1	✓	✓		
AT4G29060	EMB2726	✓			✓
AT5G20920	EIF2 beta			✓	✓
AT1G04170	EIF2 gamma			✓	✓
AT2G45730	TRM6			✓	✓

TPR proteins

AGI	TAIR alias	*in vivo (large-scale)* *GRP7 5'UTR_1 LNA*	*in vivo (large-scale)* *GRP7 5'UTR_1 LNA + LNA2.T*	*in vitro* *5'UTR*	*in vitro* *3'UTR*
AT1G02150	CCR16	✓		✓	✓
AT1G55890	MS78	✓		✓	✓
AT3G13160	MS79	✓		✓	✓
AT4G36680	ML103	✓		✓	✓
AT1G17760	CSTF77			✓	✓
AT3G13150	-	✓		✓	✓
AT5G28740	-			✓	✓

Poly(A)-binding proteins

AGI	TAIR alias	*in vivo (large-scale)* *GRP7 5'UTR_1 LNA*	*in vivo (large-scale)* *GRP7 5'UTR_1 LNA + LNA2.T*	*in vitro* *5'UTR*	*in vitro* *3'UTR*
AT4G34110	PAB1	✓		✓	✓
AT2G23350	PAB4	✓		✓	
AT1G49760	PAB8	✓		✓	✓

Splicing factors

AGI	TAIR alias	*in vivo (large-scale)* *GRP7 5'UTR_1 LNA*	*in vivo (large-scale)* *GRP7 5'UTR_1 LNA + LNA2.T*	*in vitro* *5'UTR*	*in vitro* *3'UTR*
AT1G23860	RSZ21			✓	✓
AT4G31580	RSZ22	✓		✓	✓
AT2G24590	RSZ22A			✓	✓
AT4G25500	RS40			✓	✓
AT5G52040	RS41	✓		✓	✓
AT5G06160	ATO	✓		✓	
AT2G47640	SMD2A	✓		✓	
AT5G44500	SMBA	✓			✓
AT1G03330	LSM2			✓	✓
AT1G07910	AtRLG1			✓	✓
AT2G47580	U1A			✓	✓
AT3G55460	SCL30			✓	✓
AT3G13570	SCL30A			✓	✓
AT4G36690	ATU2AF65A			✓	✓
AT3G18790	-	✓		✓	✓
AT4G21660	-	✓		✓	✓
AT5G64270	-			✓	✓

Small RNA-related proteins

AGI	TAIR alias	*in vivo (large-scale)* *GRP7 5'UTR_1 LNA*	*in vivo (large-scale)* *GRP7 5'UTR_1 LNA + LNA2.T*	*in vitro* *5'UTR*	*in vitro* *3'UTR*
AT1G48410	AGO1	✓		✓	✓
AT1G31280	AGO2			✓	✓
AT2G27040	AGO4	✓		✓	
AT2G27100	SE			✓	✓
AT2G38770	MAC7	✓		✓	✓
AT1G07360	MAC5A	✓		✓	

Additional file 11: **Overview of additional groups of proteins identified by *in vivo* and *in vitro* pulldowns of *AtGRP7* interactors.**
