# Supplementary material for: Revealing the Arabidopsis AtGRP7 mRNA binding proteome by specific enhanced RNA interactome capture
Source: BMC Plant Biol. 2024 Jun 14;24:552. doi: 10.1186/s12870-024-05249-4 (PMC11177498; doi:10.1186/s12870-024-05249-4)
Supplement: Supplementary file 12 — Supplementary Material 12 [file 12870_2024_5249_MOESM12_ESM.pdf]

Additional file 12

(A)

DNA probe #	Sequence (5'-> 3')	Probe position	Length	% GC
**1**	TTGGGGACATATCACGTGAC	24	20 nt	50.0%
**2**	CGATGTAGACGAAGTCTCGT	159	20 nt	50.0%
**3**	CAACGAAGCACCGATACTCA	259	20 nt	50.0%
**4**	CCAGTCTCACGATCGTTAAT	359	20 nt	45.0%
**5**	GTTAACAGTGATGCTACGGC	468	20 nt	50.0%
**6**	ACCACCTCTTGAGGAGTAAC	651	20 nt	50.0%
**7**	AAAGGAATTACCATCCTCCA	760	20 nt	40.0%
**8**	CATTACGTTTCCAGATACCA	908	20 nt	40.0%
**9**	CGGTATCCGATTAGCATTTC	1009	20 nt	45.0%
**10**	GGTTTCTACTTCATTGTTGA	1129	20 nt	35.0%

(B)

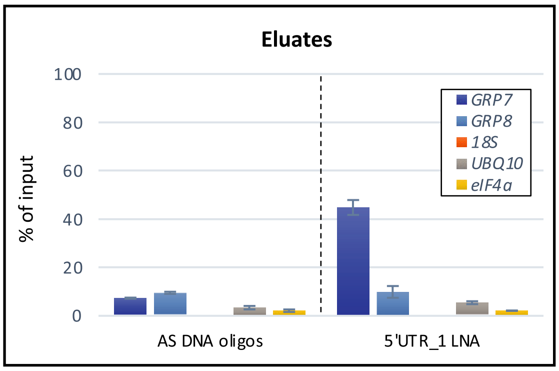

(C)

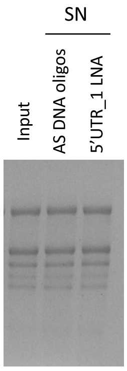

Additional file 12: Capture with antisense DNA oligonucleotides.

(A) Sequences of antisense (AS) DNA oligonucleotides directed against *AtGRP7*. **(B)** Relative *AtGRP7*, 18S rRNA, *UBIQUITIN 10*, and *eIF4a* RNA levels in the eluates of *AtGRP7*-GFP *grp7-1* plants upon capture with AS DNA oligonucleotides or with the 5'UTR_1 LNA oligo. **(C)** Agarose-formaldehyde gel electrophoresis of total RNA in the lysate (input) and the supernatant (SN) after hybridization with AS DNA oligonucleotides or the 5'UTR_1 LNA oligo in *AtGRP7*-GFP *grp7-1* plants.
